# Do Conflicts Influence the Accumulation of Bonding, Bridging, and Linking Social Capital? Insights From Cameroon

**DOI:** 10.1111/cars.70003

**Published:** 2025-03-04

**Authors:** Roland Azibo Balgah, Emmanuel Yenshu Vubo, Sirri Eunice Neba

**Affiliations:** ^1^ Department of Agribusiness Technology, College of Technology University of Bamenda Bambili Cameroon; ^2^ Faculty of Humanities, Department of Social Anthropology Sol Plaatje University Kimberely South Africa; ^3^ Faculty of Social and Management Sciences University of Buea Buea Cameroon

**Keywords:** bonding, bridging, conflict, linking, weak ties, attachement, accointance, conflit, liaison, liens faibles

## Abstract

Social capital is known to influence livelihoods, but how this operates in conflict situations is relatively under‐researched. Leaning on the social capital theory, we investigate the association between conflict and the dynamics of bonding, bridging and linking social capital in the neglected “Anglophone” conflict between a separatist movement and the government of Cameroon, which impacts livelihoods and social relations. Using data generated through mixed methods, the study explores Granovetter's concept on the strength of weak ties in a conflict context. Results reveal an overall negative causal link between conflict and social capital accumulation with significant changes in membership in social networks. Bonding social capital was comparatively less affected, while bridging and linking social capital were observed to have deteriorated. The argument is that degraded bridging and linking social capital are destructive of social relations and livelihoods, and linking social capital does not constitute strength in weak ties.

## RÉSUMÉ

On sait que le capital social influence les moyens de subsistance, mais la façon dont il agit dans les situations de conflit est relativement peu étudiée. En nous appuyant sur la théorie du capital social, nous étudions l'association entre les conflits et la dynamique des capitaux sociaux de type bonding, bridging et linking, dans le cadre du conflit « anglophone » négligé entre un mouvement séparatiste et le gouvernement du Cameroun, qui a un impact sur les moyens de subsistance et les relations sociales. En utilisant des données générées par des méthodes mixtes, l'étude explore le concept de Granovetter sur la force des liens faibles dans un contexte de conflit. Les résultats révèlent un lien de causalité globalement négatif entre le conflit et l'accumulation de capital social, avec des changements significatifs dans l'appartenance aux réseaux sociaux. Le capital social de liaison a été comparativement moins affecté, tandis que les capitaux sociaux de connexion et de liaison se sont détériorés. L'argument est que la dégradation du capital social de liaison et d'accointances est destructrice des relations sociales et des moyens de subsistance, et que le capital social d'accointances ne constitue pas une force dans les liens faibles.

## 1 Introduction

Despite lacking a unified definition, social capital has attracted enormous scholarly attention across a wide disciplinary spectrum, especially in the last four decades. Its relevance resonates from the multiple disciplines responsible for grounding it, particularly sociology (Bourdieu 1986; Coleman 1988; Putnam 1993; Azad and Pritchard 2023), economics (Woolcock and Narayan 2000; Naher et al. 2022), and political science (Putnam 1993, 1995, 2000). The concept of social capital is widely applied in studies on livelihoods (Mukisa et al. 2020; Xiong et al. 2021; Balgah and Yenshu 2024; Mahato and Jha 2024), natural resource management (Ostrom 1990; Ballet et al. 2007; Musavengane and Simatele 2016; Kimengsi et al. 2022), climate variability, change and adaptation (Pandey et al. 2018; Masud‐All‐Kamal et al. 2021; Ankrah et al. 2023), disaster management (Edoun et al. 2015; Melo‐Zurita et al. 2018; Kumari and Frazier 2021), community development (Nursey‐Bray et al. 2022), and public policy (Bezanson 2006).

The application of social capital in multiple contexts and disciplines renders a consensual definition elusive. What constitutes social capital, how it is accumulated and measured, and the extent to which it influences the accumulation of other forms of capital remains fuzzy (Bhandari and Yasunobu 2009). Advancements with the concept have been marred by divergences in the topical literature, particularly with respect to definition and measurement (Colletta and Cullen 2002). Nevertheless, social capital (SC) theorizing—which we briefly revisit later in this contribution—has generated working definitions which seem to guide research at least in the sociology, economics and political science disciplines. SC is generally understood as the norms and networks of social cooperation (Patulny and Svendsen 2007), even if questions on what constitutes them in practice persist.

One field that has not attracted sufficient social capital research relative to its contemporary relevance is conflict studies. The world is currently witnessing the highest number of violent conflicts since the Second World War, affecting 2 billion people worldwide (UN 2023). Over 117 million persons were forcibly displaced by conflicts in 2023, about one‐third of them (over 40 million) in Africa (UNHCR 2024). Over 96% of conflict victims in Africa are internally displaced persons (IDPs) (ACSS 2024). Fifty‐five state‐based and 82 non‐state conflicts were recorded globally in 2022, the highest registered for any year since 1994 (OCHA/Relief web 2023).

The surging number of (violent) conflicts in the last two decades has stimulated a growing scholarship on the conflict‐social capital nexus (Bellows and Miguel 2009; Deng 2010; Schindler 2010; De Luca and Verpooten 2011; Kiboro 2017; Calvo et al. 2019; Bate et al. 2023). With the exception of a few very recent studies (e.g., Gobbers 2024; Woldehanna et al. 2024; Igreja et al. 2025), very little knowledge exists on conflict‐social capital dynamics, particularly in contexts of neglected conflicts in Africa.^i^ Studies that disentangle the dynamics of bonding, bridging and linking social capital are scarce.

Neglected conflicts present unique contexts for analyzing social capital dynamics, given that victims do not have sufficient international support (e.g., through mandated humanitarian relief) and therefore would heavily rely on existing endogenous resources, including social capital, to *get along* (or cope) with conflict effects and eventually *get ahead*, recover, bounce back, or adapt. Dissecting SC along bonding, bridging, and linking dimensions can provide insights on the availability and contribution of intra‐community (bond), inter‐community (bridge), and cross‐institutional cooperation (link) in conflict settings.

We contend that in conflict settings, bonding SC facilitates access to resources required for short‐term coping (*mitigation*), such as food and shelter essential to reduce immediate suffering. Bridging SC provides access to resources outside immediate networks, needed for long‐term coping (*response*), such as hosting IDPs by friends and relatives in neighboring communities. Linking SC accumulated, for instance, through connections established with government, national, and international institutions out of the community, can have long‐term recovery or resilience‐building effects. Therefore, bonding SC supports victims to *get along*, bridging allows them to *get by*, and linking SC to *go ahead* in conflict situations (Putnam 2000). The level of accumulation of all three SC forms influences victims’ capacity to *get back*, recover, or bounce back. Gobbers (2024), for instance, demonstrates that bonding SC reinforced intra‐group solidarity, while bridging ties broadened solidarity in some communities in war‐torn Democratic Republic of Congo. These, in combination with linking social capital, mitigated inter‐group conflicts. In contrast, Igreja et al. (2025) contend that violent conflicts negatively affected family relations and social networks during the civil war in Mozambique. By fragmenting collective identities, the conflict reduced collective action in pre‐ and post‐conflict Mozambique. Divergence in empirical outcomes of the role of social capital in conflict situations justifies further research.

We extend the notions of bonding, bridging, and linking SCs to empirically analyze the effects of conflict on SC dynamics among persons internally displaced by the sociopolitical conflict in the two Anglophone (North West and South West) regions of Cameroon. Additionally, we examine the role of linking SC and the ability of conflict victims to recover based on the notion of the strength of weak ties (Granovetter 1973, 1983; Putnam 2000; Skobba et al. 2018).

The following research questions guide the study:

1. What dynamics in bonding, bridging and linking social capital are observable in the context of neglected conflicts in Africa?
2. Do these dynamics reflect the notion of the strength of weak ties?

In line with SC theory, we hypothesize that bonding and bridging capital are more relevant in the early years (or phase) of conflicts, where getting along and getting by are more important than getting ahead. Much in line with the strength of weak ties argument, we also expect that linking SC will be largely static due to limited international support that would otherwise nurture its growth. Static or slow‐expanding linking SC might impede rapid recovery among victims.

As will be demonstrated, our study revealed an overall negative causal link between conflict and SC accumulation, albeit with differences between SC types. While bonding SC was more robust, bridging and linking social capital deteriorated as a result of the conflict. Diminishing bridging and linking social capital are likely to retard socioeconomic recovery in neglected conflict situations. Understanding conflict‐social capital dynamics can provide an explanation as to why some conflict victims recover rapidly in conflict settings and not others.

The article makes a number of contributions to the study of social capital. First, it adds to the very limited SC‐linked studies on conflicts, with an empirical case study from Cameroon. The Cameroon case study is particularly interesting, given that it constitutes the second most important neglected conflict in the world (NRC 2024). This case study can provide relevant insights for managing neglected conflicts and generate a framework for further analysis of similar conflicts in Africa, which hosts 90% of all globally neglected conflicts. Second, it contributes empirical data to validate the contentions of social capital theory encapsulated in discourses on the strength of weak ties, namely, that weak ties leverage more cohesive power than strong ones (Granovetter 1973).

## 2 Theoretical Framework: An Overview of Social Capital Theory

Although the historical roots of social capital are linked to concepts such as civic engagement and social stratification (van Bakel and Horak 2024), contemporary social capital theory has been influenced mainly by contributions of seminal theorists from economics, sociology, and political sciences. Three scholars with diverse disciplinary backgrounds—Pierre Bourdieu, James Coleman, and Robert Putnam—are generally credited for key advances with social capital theory (Engbers et al. 2017; Claridge 2018; van Bakel and Horak 2024).

Bourdieu (1986) systematically differentiated economic from cultural, social, and symbolic capital. The fundamental goal of his thesis was to demonstrate the relevance of social capital as an additional perspective to the economic notion of self‐interest in understanding the economy of practice (Bourdieu 1986; Bourdieu et al. 1993). He distinguished between economic capital, linked to money and institutionalizable in property rights, and cultural capital, convertible to economic capital under certain circumstances, and institutionalized in the form of qualifications. He construed SC as social obligations and connections, which under certain conditions can be converted into economic capital (Bourdieu 1986; Tsounis and Xanthopoulou 2024). Consequently, Bourdieu (1986) defined social capital as an aggregate of actual and potential resources, linked to possession of a durable network of more or less institutionalized relationships of mutual acquaintances. Bourdieu's SC theorizing has been criticized for focusing on the individual and only considering collectivities in the notion of cultural capital.

Contrary to Bourdieu, Coleman (1988) integrated economic rational theory into social systems analysis. He did this by linking social action to social structure (a functionalist view) and by contending that individual actions are driven by a self‐interest rationality (Coleman 1988). Given the rational nature of humans, they will engage in social interactions, relationships and networks as long as benefits from these accrue to them—a logic that is also maintained in social organization (Claridge 2018). In this sense, SC is both an individual and collective good whose relevance is contingent on the benefits it provides for its owners. Coleman (1988) conceptualized SC as the relations among persons that facilitate cooperation between people in pursuit of mutual objectives. Coleman is lauded for bridging the theoretical gap between economics and sociology, for stimulating cross‐disciplinary research (van Bakel and Horak 2024), and for defining SC by scope and function (Engbers et al. 2017).

Emerging from a civic engagement political science perspective, Putnam (1993) conceptualized SC as features of social organization: “norms, networks and trust that facilitate action and cooperation for mutual benefit” (p.35). By specifying measurable components of SC and the consequences of its absence, Putnam laid the foundation for an aggregate measure of SC and for scientifically exploring related cause‐effect relationships (Claridge 2018; van Bakel and Horak 2024).

Despite these divergences in SC theorizing, there seems to be consensus that (1) SC capital exists, (2) it is expressed in relationships and networks, and (3) it can be leveraged for individual or collective benefits. SC can take several forms; it can be structural, cognitive, or embedded (Bhandari and Yasunobu 2009; Igreja et al. 2025). It can also be conceptualized as a bond or a bridge (Putnam 2000; Szreter and Woolcock 2004), or a link (Granovetter 1973, 1983), and it can have both positive and negative effects (Claridge 2018; Tsounis and Xanthopoulou 2024).

### 2.1 Bonding, Bridging, and Linking Social Capital

Bonding, bridging, and linking SC are potentially the most relevant entrants in contemporary SC theory (Szreter and Woolcock 2004). Bonding SC comprises relations of trust and cooperation among people with a shared social identity. Examples include relations between people of the same class or family (Szreter and Woolcock 2004; Geys and Murdoch 2010) and among friends. Bridging SC comprises relations of respect and mutuality between unlike people across diverse social categories such as age, ethnicity, or class (Putnam 2000; Szreter and Woolcock 2004) and membership in groups and networks (Geys and Murdoch 2010). Linking SC is defined as the norms and networks of trust between people who are interacting across explicit institutionalized power or authority boundaries in society (Putnam 2000; Szreter and Woolcock 2004), such as between communities and social, economic and political institutions (Woolcock 1998; Patulny and Svendsen 2007). Linking SC leans on generalized trust between two largely unfamiliar entities (Grootaert et al. 2004; Azad and Pritchard 2023), and it is this (weak) link that is hypothesized to generate the highest benefits to its members (Granovetter 1973, 1983).

The quality and quantity of bonding, bridging, and linking social capital—all strongly contingent on trust levels—influence the effects of SC on economic and social outcomes (Szreter and Woolcock 2004). Empirical studies have validated this hypothesis, for instance in explaining differences in economic returns for migrants to the Netherlands and Germany (Lancee 2010, 2012), in livelihood differences among people residing in low income neighborhoods in the United States (Brisson and Usher 2007), in health outcomes among Chinese adults (Chen and Meng 2015), and in community outcomes through volunteerism (Nursey‐Bray et al. 2022). With a few exceptions (Gobbers 2024; Igreja et al. 2025), case studies that explore the dynamics of bonding, bridging, and linking SC dynamics in conflict contexts are extremely difficult to find. We narrow this knowledge void by examining changes in bonding, bridging, and linking SCs among people affected by the Anglophone conflict in in Cameroon's North West and South West regions.

Contemporary literature on the Anglophone conflict in Cameroon largely focuses on themes that include the causes and nature of the conflict (Nwati 2020, 2021; Nyadera et al. 2020), its historical origins (Konings and Nyamjoh 1997, 2019; Pelican 2022), its capacity to trigger complex emergencies (Bang and Balgah 2022), and the role of social media in the conflict (Nkongho 2018). Emerging cases focusing on SC (e.g., Bate et al. 2023) have not properly studied bonding, bridging, and linking SC dynamics, which are likely to shape endogenous capacities to get along, get by, and go ahead (Gobbers 2024). Our study draws from an active conflict to improve the understanding of the conflict‐SC nexus for which scholarly effort is less visible. We do this by empirically analyzing household bonding, bridging, and linking SC dynamics before and during the conflict, specifically between 2015 and 2021.

## 3 Data and Methods

### 3.1 Study Area

Cameroon has witnessed several violent conflicts in recent years. The two most important contemporary ones include the Boko Haram insurgency in the northern regions of Cameroon, and insurgency in its North West and South West (Anglophone, predominantly English‐speaking) regions (Bang and Balgah 2022; Pelican 2022). The impacts of conflicts on SC, divided communities, or undermined social cohesion among households, remain largely unknown. Gaining a better understanding of these issues is relevant for conflict management policy making and implementation.

The Anglophone conflict is rated as the second most neglected armed conflict in the world (NRC 2024) and the newest armed conflict in Central Africa (Tanyu 2022). The conflict is largely rooted in Cameroon's complex pre‐ and post‐colonial history, particularly in the linguistic and cultural incongruences emanating from long periods of separate British and French colonial rules in the former Southern (English‐speaking) and French (French‐speaking) Cameroons, respectively.

Cameroon was a German colony from 1884 to 1916, after which it was shared by the British (20%) and the French (80%) as an outcome of the 1919 Versailles treaty (Konings and Nyamnjoh 1997, 2019). As a result, this former German colony became a bi‐cultural and bi‐lingual British and French colony (Bang and Balgah 2022). In January 1960, the French‐colonized Cameroon gained independence as the Republic of Cameroon. In 1961, British‐colonized Cameroon agreed to join the independent Republic of Cameroon to form a Federal Republic of Cameroon (Konings and Nyamnjoh 1997, 2019).

Skepticism of this reunion between the two Cameroons arose in 1972, when the pioneer President H. E. Amadou Ahidjo changed the country's name from Federal to United Republic of Cameroon, on the grounds of a plebiscite (Pelican 2022). This skepticism peaked in 1984, when the incumbent President H. E. Paul Biya changed the country's name (back) to the Republic of Cameroon, which in the *senso stricto* was the original name for independent Cameroon. These changes, which some saw as anti‐constitutional (Konings and Nyamnjoh 1997; Pelican 2022), bred discontent and contributed to fears of assimilation and marginalization among the predominantly English‐speaking (Anglophone) regions, who consistently used peaceful means such as ghost towns (*‘villes morte*), protests, calls for restoration of the Federal state (e.g., through resolutions of multiple All Anglophone Conferences in the 1990s), and petitions to the United Nations to raise their concerns about the changing forms of the state (Konings and Nyamnjoh 2019). Unfortunately, these grievances were never given full attention.

Contemporary violence started with a protest by teachers’ and lawyers’ trade unions in Cameroon's Anglophone (predominantly English‐speaking, North West and South West) regions in 2016 (Nyadera et al. 2020; Pettersson and Oberg 2020; World Bank 2021). The unions were protesting what they considered to be the persistent marginalization of Anglophone linguistic, cultural, and educational traditions and systems, and deliberate, weak Anglophone representation in politics (Amnesty International 2023). It later degenerated into confrontations between the military and separatist groups seeking independence for the two Anglophone regions.

Since violence started in 2017, about 598,000 people have been internally displaced, and roughly 2 million are in need of humanitarian aid (HRW 2022). An estimated 6500 deaths have occurred, mainly due to clashes between the military and separatist groups (ICG, 2024). Internal displacement has generally been from rural areas where fighting is intense into urban cities in the same regions, when these cities are considered safer, or into other regions not directly affected by the conflict. As the conflict is categorized as neglected (NRC 2024), we contend that conflict victims will highly depend on their endogenous resources—including their social capital—to deal with some conflict consequences, especially those that lead to dysfunctions, given limited international visibility and external humanitarian support. It then becomes interesting to analyze bonding, bridging, and linking SC dynamics among IDPs in the conflict zones and the extent to which these dynamics succumb to the strength of weak ties notion.

This empirical study was carried out in Kumba, the capital of the Meme division and the largest town in the country's South West region (SWR). The population of Kumba has grown from about 144,000 in 2015 to 225,000 in 2020 (a 56.3% increase), mainly due to a considerable influx of IDPs of the Anglophone conflict (World Bank 2021). Its centrality and nodal point in the economic, political, and social landscapes have made Kumba an internal refuge town for migrants from the SWR and other regions fleeing from the conflict. It has a population density of 51.8 inhabitants per square kilometer. Seventy‐five percent of the population falls within the youthful age group (Akoachere and Ngwese 2017).

As a cosmopolitan town, Kumba hosts people from other countries like Nigeria, Chad, Benin, and Ghana, who cohabit peacefully with indigenes. Kumba is a key economic hub in the region, in addition to being an important site for the creation and sustenance of community‐based organizations which enhance SC accumulation amongst its members (Tiafack and Ngouanet 2008). The changing demography, involuntary displacements, rising insecurity, and violence observed during the Anglophone conflict between 2016 and 2021 make Kumba a unique case study to explore how conflict influences SC accumulation.

### 3.2 Study Design

Mixed methods were employed in a cross‐sectional survey to collect data on changes in different (bonding, bridging, and linking) SCs from persons (household heads) affected by the conflict and residing in the three subdivisions of Kumba City, that is, Kumba I, II, and III, as part of a comprehensive study on the impact of conflict on household livelihoods in the study area. These three subdivisions have witnessed large demographic and social changes since violence was ignited in 2016, presenting favorable contexts for SC analysis. Affected household heads were targeted, since their authority and responsibility render them best placed to provide information at the household level.

Quantitative data was obtained through the administration of a structured, pre‐tested questionnaire to randomly selected household heads, with the support of six trained research assistants. Drawing from existing SC literature (Putnam 2000; Grootaert et al. 2004; Szreter and Woolcock 2004; Patulny and Svendsen 2007; Geys and Murdoch 2010), bonding SC was measured using within‐community family and friendship relations, and bridging SC using social interactions and membership in groups and diverse social networks, both outcomes of which are contingent on existing trust levels. Linking SC was proxied by generalized trust across independent networks and institutions (see Table 1). Responses were captured between two time slots: 2015 (with no conflict) and 2021 (with conflict) using recall, due to the absence of any kind of recorded data.

**TABLE 1:** Summary of variables used in the study by social capital type.

Form of social capital	Fundamental variables	Operational variants relevant in the conflict situation
Bonding	Friendship	Number of close friends in the community
	Family relations	Assistance from kin‐group based relations
Bridging	Social interactions	Assistance to household from friends and relatives (local networks)
	Group membership	Number of groups/associations to which household members belong
Linking	(Inter)Community level Generalized Trust	Generalized trust across independent networks and institutions

*Source*: Adapted from Putnam (2000), Grootaert et al. (2004), Szreter and Woolcock (2004), Patulny and Svendsen (2007), Geys and Murdoch (2010).

### 3.3 Sampling and Sample Characteristics

The method of determining the sample size for quantitative data collection using the structured questionnaire followed the following Taro Yamane sampling formula:

(1) $$n=N/\left(1+N{\left(e\right)}^{2}\right)$$

where *n* refers to the target population; *n* is the sample size; and *e* is chosen margin of error (0.05). Therefore, sample size is 8285/(1+8286(0.05)^2^) = 400.

Respondents were randomly selected from IDPs in all three subdivisions (Kumba I, II, III) using a ratio of 9:6:5. The ratio reflects the fraction of the population that had experienced the ongoing conflict, as reported by key informants during the reconnaissance visit to the communities prior to the study. Sampling frames of IDP households were developed in each subdivision with the help of local leaders and used as the basis for simple random sampling of interviewees. Randomization was terminated when the target for each subdivision was met. The final sample for the quantitative survey (400 respondents) included 180 respondents in Kumba I, 120 in Kumba II, and 100 in Kumba III.

### 3.4 Data Collection and Analysis

Data was collected at the household level with the help of six enumerators who were recruited and trained. All participated in questionnaire pre‐testing during the contact visit to the targeted communities. Interviews took place at the homes of respondents, except when this was not possible, either due to the commitment of the household head or insecurity.

Qualitative data was obtained through in‐depth interviews and focus group discussions, which are effective in exploring personal experiences, perceptions, and perspectives (Kivale and Brinkman 2009). In‐depth interviews were carried out with selected male and female household heads (*n* = 9) based on their personal experiences with the conflict. Selection was done during the quantitative data collection process. Only household heads who gave their verbal consent (by agreeing to participate) and who had been directly affected by the conflict, for instance through forced displacement, kidnapping, threats of life, loss of family member, or direct attacks, were selected for in‐depth interviews.

Two focus group discussions (FGDs) were conducted with nine purposively selected household heads in each group, disaggregated into female‐ and male‐headed households (55% women and 45% men). FGDs explored and captured individual and collective experiences and perspectives on nature, the evolution, and dynamics of the conflict, the impact on livelihoods, livelihood strategies, coping mechanisms, and aspects of bonding, bridging, and linking SCs. FGDs provided detailed information that could not be captured during the survey. Overall, qualitative data collection provided insights that enriched the interpretation of quantitative data. In total, 427 respondents took part in the study. Data collection took place between April 1, and May 31, 2022.

Primary data was complemented with information from secondary sources obtained from literature searches on SC and conflict. Quantitative data was analyzed by performing descriptive statistics on selected bonding, bridging, and linking SC variables for the two time slots (2015 and 2021) and discussed adopting the 5% level of significance. Qualitative data was used to better explain the quantitative results.

## 4 Findings and Discussion

### 4.1 Descriptive Statistics

The study sample was 62% male and 38% female. This gender bias is explained by the dominance of patriarchal systems in the study region that favor male household heads over female ones (Balgah 2016). Education levels were found to be high among respondents, as only 2% had no formal education. About 17% had completed primary school, 29% secondary school, 51% post‐secondary school, and 1% university studies. This points to—from an educational point of view—very high human capital among respondents. The mean sample age of 45 years (45.29 ± 12.394) is higher than Cameroon's current mean age of 18.5 years (Cameroon Population Clock 2022; Worldometer 2024). This is expected, given that interviews were conducted with household heads.

The mean household size is 8 with a minimum of 2 and a maximum of 32. This is higher than Cameroon's national average of 5, or 6 for the study area before the violent phase of the conflict erupted (Balgah 2016). Large household sizes probably result from forced (internal) displacement. Large household sizes increase the probability of networking for SC accumulation (Yaya 2009; Deng 2010). However, with a mean of 4 children per household, this is not automatic, as children may not have the capacity to create and/or sustain durable networks.

### 4.2 Social Capital Dynamics in Conflict Situation

This section investigates the changes observed in households’ SC within the two time slots. We examine any changes for bonding, bridging, and linking SCs, using context‐specific variables (see also Bate et al. 2023), which generally match standard measurement variables drawn from the topical literature (Putnam 2000; Grootaert et al. 2004; Szreter and Woolcock 2004; Azad and Pritchard 2023).

### 4.3 Bonding Social Capital

#### 4.3.1 Number of Close Friends in Local Community

Table 2 presents the mean number of friends reported by household heads affected by the conflict in the city of Kumba in Cameroon. The mean number of friends per respondent significantly dropped from 3 to 2 between 2015 and 2021. Given that strong bonding motivates norms of reciprocity and solidarity among family and friends, for instance, through the provision of food and shelter (Azad and Pritchard 2023), bonding SC de‐accumulation in the Cameroon case may weaken endogenous coping capacity. Decreasing friendship ties were attributed to increasing distrust among friends, which in the best scenario weakens friendship networks but may also destroy them completely (Jasper and Shoham 2002; Igreja et al. 2025). As one of the participants of the in‐depth interviews said:

“This thing [the conflict] is very political, and many people are blacklegs [sell‐outs]. No one, not even close friends and relatives can be fully trusted” (IDI 24.04.2022).

In other words, the conflict created a divide between those who are for or against separation, leading to deepening distrust between friends and neighbors. This statement also suggests that a drop in bonding SC captured through the number of friends between 2015 and 2021 in the study areas is at least partially attributable to the ongoing conflict.

**TABLE 2:** Number of close friends.

Bonding social capital variable	Year	Mean	Std. Deviation	*t*‐distribution
Number of close friends within the community	Before conflict (2015)	3	6	*t* = 1.905^*^
	With conflict (2021)	2	4	

*Source*: Own empirical data (2022).

*Note*: ^*^ *p* < 0.05, ^**^ *p* < 0.01, and ^***^ *p* < 0.001.

Figures have been rounded to the nearest whole number.

#### 4.3.2 Local Sources of Household Assistance

**FIGURE 1:**
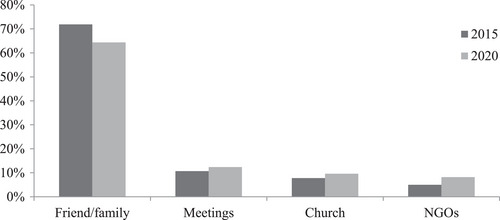
Source of assistance to household from networks. *Source*: Own empirical data (2022). *Note*: 2015 is the period without conflict and 2020 is within the conflict.

When questioned on the sources of households’ assistance in 2015 (pre‐conflict) and in 2021 (during the conflict), most respondents reported receiving assistance from a friend or family member in 2015 and 2021. However, the rate of receiving assistance from a friend or family member dropped by 7.5% points between the two time points, from 71.9% in 2015 to 64.4% in 2021 (Figure 1). This assistance was in the form of food (32.4% in 2015 and 34% in 2021), financial support (27% in 2015 and 26.7% in 2021), clothing (4.6% in 2015 and 34% in 2021), agricultural information (5.4% in 2015 and 6.1% in 2021), and psychosocial support (2.3% in 2015 and 5.2% in 2021). That strong bonds support access to basic needs lends credence to previous studies (van Bastelaer 2003; Bate et al. 2023; Azad and Pritchard 2023). In fact, access to basic needs such as food and shelter during crises can provide therapeutic benefits to those suffering (Coleman 1988; Narayan and Pritchett 1997; Grootaert et al. 2004), as was observed in the Cameroon case study.

Bonding SC remains a major source of livelihood assistance, despite the drop between 2015 and 2021. This suggests that households increasingly depend on bonds as a last resort. Such networks are likely to be overburdened, and in the worst scenario ineffective, leading to livelihood deterioration among IDPs, who in neglected conflict situations may not have adequate access to external humanitarian support.

Slight increases in humanitarian assistance were observed among local self‐help groups (known locally as ‘meetings’) over the same period (from 10.7% in 2015 to 12.4% in 2021), from churches (from 7.8% in 2015 to 8.9% in 2021), and local NGOs (from 5% to 8%). Overall, over 95% and 92% of all sources of assistance before and during the conflict, respectively, originated from endogenous, community‐based SC resources, as community‐based networks became the most important and probably the only reliable safety nets for conflict victims (Aghajanian 2012; Gobbers 2024). In this context, bonding SC can be considered to be resilient to the conflict.

### 4.4 Bridging Social Capital

#### 4.4.1 Membership in Social Groups and Networks

The study findings show that most households within the study area belong to social groups and networks, with little or no change in membership over time (82% in 2015 and 81.3% in 2020, *X* ^2^ = 2.045, *p* = 0.843). This is an indication of the relevance of social groups and networks in the context of neglected conflicts. Previous studies have shown that belonging to groups and social networks leverages resources that members need in order to survive perverse events, such as natural disasters (Balgah and Buchenrieder 2010, Dressel et al. 2020; Azad and Pritchard 2023), and political conflicts (Bate et al. 2023; Igreja et al. 2025). Working with endogenous community groups and networks can potentially reduce the livelihood burden for IDPs of neglected violent conflicts.

Although membership in groups and/or networks is relevant, the density (or number) of membership in networks provides the holder real advantages (Grootaert et al. 2004). Network density determines access to actual and potential resources (Bourdieu 1986) and to collective action and cooperation (Putnam 1995), which in turn facilitate the attainment of individual and collective goals (Narayan 1999; Igreja et al. 2025). To capture this component, we analyzed changes in group membership before and during the conflict. The results (Table 3) indicate a significant drop, from a mean of 2 groups in 2015 to 1 in 2021 (*t* = 2.572, *p* = 0.01). Thus, although membership in groups and networks is high, actual SC that accrues to members may be negatively affected by the studied conflict. This drop is probably due to soaring insecurity, violence, and restricted movements that have accompanied the crisis (Bang and Balgah 2022; Pelican 2022; Bate et al. 2023). In addition, membership in social groups often demands financial engagements that affected persons might find difficult to provide in crisis situations. These results are consistent with the conclusions of some studies (e.g., De Luca and Verpoorten 2011) but do not confirm other studies (e.g., Yaya 2009; Calvo et al. 2019) that report an increase in group membership during conflict.

**TABLE 3:** Membership in groups and associations.

Bridging social capital variable	Year	Mean	Std. Deviation	*t*‐distribution
Number of associations (groups and networks) household members belong to	2015	2	1	*t* = 2.572^**^
	2021	1	1	

*Source*: Own empirical data (2022).

*Note*: ^*^ *p* < 0.05, ^**^ *p* < 0.01, and ^***^ *p* < 0.001.

#### 4.4.2 Social Interaction

Social interaction indicates the level of social capital in a community. Tight‐knit relations within kin groups (e.g., family) suggest the strength of bonding, while interactions beyond this (e.g., with neighbors and community members) are more indicative of bridging social capital (Grootaert et al. 2004). Respondents were asked who they interact with on a regular basis before and during the conflict, and if this interaction changed between the two time slots. Results are presented in Table 4. Interactions with the family significantly increased by 6.1% points between 2015 and 2021. However, interaction with friends and community members (bridging) dropped by 3.8% and 2.3%, respectively, over the same period, compared to pre‐conflict times. That households lean more on immediate family to get along than on more distant relatives and friends and community relationships suggests that getting along is prioritized over getting by in the Cameroonian case, where distrust is omnipresent. Hardship resulting from restrictions on activities going by the name of “lockdowns” or “ghost towns” imposed by separatists also strengthens bonding in kinship groups (close‐knit family ties) at the expense of bridging (community‐level) relations (Bate et al. 2023). In fact, the conflict in the Anglophone regions of Cameroon has been characterized by blackmail, payback, victimization, settling of scores, and violence (Nwati 2020, 2021).These factors instigate fear and distrust in the affected population, both of which are catalysts of SC breakdown.

**TABLE 4:** Social interaction.

Variables Bridging social capital variable	Year	Immediate family	Relatives and friends	Community members	Chi‐square
Who respondents interact with	2015	70.4%	24.7%	4.9%	*X* ^2^ = 15.975^**^
	2021	76.5%	20.9%	2.6%	

*Source*: Own empirical data (2022).

*Note*: ^*^ *p* < 0.05, ^**^ *p* < 0.01, and ^***^ *p* < 0.001.

Focus group discussions revealed that community networking has been limited to essential societal activities such as funerals, as security (staying alive) takes precedence over social interaction. Conflicts that perpetuate violence and increase tension and distrust are likely to reduce cooperation beyond immediate family and friends, as division among community members increases (Aghajanian 2012). Igreja et al. (2025) reported such trends in in conflict stricken Mozambique. In this sense, we contend that the conflict seems to prioritize bonding SC at the expense of the bridging variant.

### 4.5 Linking Social Capital

Generalized trust and interactions with social and political institutions are key measures of linking SC (Putnam 1995; Grootaert et al. 2004; Azad and Pritchard 2023). In the Cameroon case, generalized trust was measured through subjective assessment of trust across independent community networks and with NGOs and political institutions outside the conflict communities.

#### 4.5.1 Generalized Trust

The results on generalized trust (Table 5) indicate a significant drop in 2021 (8.5%) as compared to trust levels in 2015 (34.5%) (26% drop, *X* ^2^ = 74.143, *p* = 0.000) across community‐based networks. In addition, over 82% of respondents did not have trust in NGOs and government‐related institutions. Similar trends have been reported in related studies (Yaya 2009; Deng 2010; De Luca and Verpoorten 2011; Naher et al. 2022).

**TABLE 5:** Level of trust in community and across networks.

Trust variable	Year	No trust	Cannot tell	Can be trusted	Statistic
Generalized trust within independent community networks	2015	35.9%	29.6%	34.5%	*X* ^2^ = 74.143^***^
	2021	55%	36.5%	8.5%	
Trust across networks and political institutions	No trust	Very little trust	Acceptable	High trust	Very high trust
	31.6	50.7	14.2	3.2	0.3

*Source*: Own empirical data (2022).

*Note*: ^*^ *p* < 0.05, ^**^ *p* < 0.01, and ^***^ *p* < 0.001.

The eroding level of trust is emphasized by a respondent in a focus group discussion:

These days walls have ears. You cannot talk anyhow because you do not know who is listening. We have freedom of speech during talking but not after talking. The best thing to do is not to discuss anything related to this struggle. (FGD, 21:04:2022)

The level of distrust is further captured in this assertion by a respondent during an in‐depth interview:

We are caught in between the dark blue sea and the lion. You cannot talk against the activities of the separatist as you would be tagged a traitor by the separatists and marked for torture or death. Neither can one side with the activities of the separatists as the military would pick you up as a supporter of terrorist activities. On the other hand, we can neither side with the military nor report the separatists to the military as the “boyses” [separatists] would tag you a “black leg” [traitor]. On either side you are going to be tortured and even killed. The best way is to remain as neutral as possible. (IDI, 18:04:2022)

Given its political nature, the Anglophone conflict is likely to divide community members based on differing political opinions. In addition, the existing social order in conflict communities has been disrupted by the activities of separatists, popularly called “Amba boys” (Amnesty International 2023), whose coercive structure and repressive approaches generate fear and distrust in affected communities (Bate et al. 2023). Increasing distrust breeds suspicion and insecurity and facilitates the breakdown of institutions, norms, and values, which culminates in deteriorating SC and social relations (Igreja et al. 2025). In our Cameroon case, decreasing trust seems to reduce linking SC accumulation in the form of collective action across independent community networks, and cooperation, with external institutions (Colletta and Cullen 2002). This significantly slows down conflict recovery (Wiggins et al. 2021; Gobbers 2024). If this logic is accepted, then the absence of linking SC seems to torpedo conflict victims’ efforts to navigate beyond getting along and getting by to going ahead, as vital weak linkages (e.g., to humanitarian assistance from specialized organizations) are missing. This seems to be the fate of victims in neglected conflicts. Further research is, however, needed to ground this contention. What seems clear in this case study is that the missing (weak) link to vital institutions significantly slows down recovery processes, thus endorsing Granovetter's notion of the strength of weak ties.

## 5 Conclusion

Although SC research has been embraced by multiple disciplines and research themes, empirical application in conflict situations, particularly with respect to bonding, bridging, and linking SC dynamics, is still in its very infancy. We add to this literature by examining the conflict‐social capital dynamics among persons affected by the Anglophone conflict in Cameroon between the quiescent, non‐conflict period (2015) and when the conflict was active (2021). Although bonding SC dropped compared to the pre‐conflict period, it remained the most resilient form of SC and was appropriated in conflict times to compensate for bridging SC de‐accumulation. This supports conflict victims to get along. Although membership in groups and networks did not change during conflict times, the density—captured through the number of networks—significantly dropped. Social interactions were largely limited to family and friends, with diminishing community interactions observed during the conflict period. Such shifts further limit bridging SC accumulation which victims need to get by. Similar trends have been reported in related studies in which conflicts were found not to significantly influence intra‐group and intra‐community solidarity (Bate et al. 2023; Gobbers et al. 2024; Igreja et al. 2025). However, unlike other studies, the number of networks and trust between independent community organizations and unfamiliar social, economic and political institutions (linking SC) significantly dropped during conflict times. Linking SC could not be explored in the Cameroon study probably due to the political nature of the conflict which seems to have bred high levels of distrust, especially with organizations and institutions external to the conflict environment.

Deteriorating linking SC failed to provide the much‐needed (weak) linkages that would have put strategic resources at the disposal of IDPs of the Anglophone conflict in Cameroon, which they need to surpass going by (coping with the conflict) to getting ahead, for instance, through recovery and reconstruction. Reduced weak links, as observed in the Cameroon (neglected) conflict, seem to be a fundamental outcome when distrust increases, making it difficult to establish fundamentally useful weak ties. In our case study, conflict victims could not explore the strength of weak ties, as suggested, for instance, by Granovetter. The current study therefore highlights the possible role of politics and the mediating role of trust in influencing access to and benefits from weak links in conflict situations. Further research is necessary to better understand the politics‐SC nexus in conflict situations and to establish if this relation is specific to neglected conflicts, as exemplified by the current case study, or generalizable.

This study brings to light a number of issues of relevance to conflict‐SC research. First, the extent of bonding, bridging, and linking SC existing prior to conflicts is likely to influence conflict outcomes. Victims can draw on bonding and bridging SC to get along and get by (cope) in the short run. However, links to unfamiliar institutions are crucial for long‐term recovery. These weak links are not quasi‐automatic, especially in neglected conflict situations with heightening levels of politically linked distrust. Second, given that the level of SC is contingent on prior trust, networks, and norms, understanding its existence, dynamics, and influence on conflict outcomes requires a context‐specific approach. More systematic research is necessary to identify trends across space and time, with the hope of developing a guiding framework for conflict‐SC research, especially for neglected conflicts.

We acknowledge the limitations of the study, particularly the limited number of bonding, bridging and linking SC variables included in the study. Including more variables in future research is likely to generate robust patterns, with respect to the conflict‐SC interactions. This will allow for more complex (multivariate) analyses as suggested for instance by Grootaert et al. (2004), or to develop composite indices that can allow for more systematic analyses on the conflict‐SC nexus. Unlike others (e.g., Craig et al. 2023), we contend that research on bonding, bridging and linking SC is more credible when the analysis focuses on each SC form as part of an interlinked capital system, in which one form of SC is not isolated from the other.

## Endnotes

i A neglected conflict is one that does not attract sufficient humanitarian and media attention and often lacks international political and diplomatic support (NRC 2023, 2024). Cameroon topped the list of countries with neglected conflicts in 2018 (NRC 2023) and is currently second after Burkina Faso. Nine of the top 10 neglected crisis in the world are currently located in Africa (NRC 2024).
